# Responses of the Insulin Signaling Pathways in the Brown Adipose Tissue of Rats following Cold Exposure

**DOI:** 10.1371/journal.pone.0099772

**Published:** 2014-06-10

**Authors:** Xiaofei Wang, Richard Wahl

**Affiliations:** The Russell H. Morgan Department of Radiology and Radiological Science, Division of Nuclear Medicine, Johns Hopkins University School of Medicine, Baltimore, Maryland, United States of America; University of Warwick – Medical School, United Kingdom

## Abstract

The insulin signaling pathway is critical for the control of blood glucose levels. Brown adipose tissue (BAT) has also been implicated as important in glucose homeostasis. The effect of short-term cold exposure on this pathway in BAT has not been explored. We evaluated the effect of 4 hours of cold exposure on the insulin pathway in the BAT of rats. Whole genomic microarray chips were used to examine the transcripts of the pathway in BAT of rats exposed to 4°C and 22°C for 4 hours. The 4 most significantly altered pathways following 4 hours of cold exposure were the insulin signaling pathway, protein kinase A, PI3K/AKT and ERK/MAPK signaling. The insulin signaling pathway was the most affected. In the documented 142 genes of the insulin pathway, 42 transcripts (29.6%) responded significantly to this cold exposure with the least false discovery rate (Benjamini-Hochberg Multiple Testing: −log10 (p-value)  = 7.18). Twenty-seven genes (64%) were up-regulated, including the insulin receptor (Insr), insulin substrates 1 and 2 (Irs1 and Irs2). Fifteen transcripts (36%) were down-regulated. Multiple transcripts of the primary target and secondary effector targets for the insulin signaling were also up-regulated, including those for carbohydrate metabolism. Using western blotting, we demonstrated that the cold induced higher Irs2, Irs1, and Akt-p protein levels in the BAT than in the BAT of controls maintained at room temperature, and higher Akt-p protein level in the muscle. Conclusion: this study demonstrated that 4 hours of cold exposure stimulated the insulin signaling pathway in the BAT and muscle of overnight fasted rats. This raises the possibility that acute cold stimulation may have potential to improve glucose clearance and insulin sensitivity.

## Introduction

Brown adipose tissue (BAT) is a thermogenic organ and consumes lipids and carbohydrates. BAT metabolic activity is increased in a cold environment [1], [2]. The unique energy consumption of stimulated BAT might be useful in controlling obesity and diabetes by modification of the body's energy balance and lead to more expenditure of energy and less deposition of fat. Over a decade ago functioning BAT was unexpectedly identified in adult humans by fluodeoxyglucose positron emission tomography (^18^F-FDG PET), a modern functional imaging modality [3], [4], [5]. Adults with FDG-avid BAT have lower body mass indices (BMI) than individuals with non-FDG avid BAT [3], [6]. Adults without FDG-avid brown adipose tissue on PET imaging had a higher risk of abnormally increased glucose levels than patients with FDG-avid brown adipose tissue [7]. This finding leads to the hypothesis that purposely stimulating BAT could have a role in controlling obesity and diabetes.

Cold temperature is a natural stimulator of BAT thermogenesis [4]. Cypess et al reported that cold activated human brown adipose tissue but sympathomimetics (ephedrine) did not [8]. In a series of rat experiments, cold exposure (48 hours at 4° or 5°C) reproducibly activated brown adipose tissue, and was associated with accelerated glucose clearance [9], [10], [11], [12].

Gasparetti et al addressed the correlation between glucose clearance and the insulin signaling pathway in brown and white adipose tissue, skeletal muscle, and liver of rats chronically exposed to a cold environment (at 4°C for 8 days) or at room temperature (23°C) [13]. They focused on initial and intermediate steps of the insulin signaling pathway using immunoblotting and immunoprecipitation. All of the tissues except the liver increased glucose uptake after the cold exposure. Only brown adipose tissue responded to the cold exposure by significantly upregulating the protein level of insulin receptor substrate 2 (IRS2). This cold condition significantly potentiated insulin-induced phosphorylation of the insulin receptor, insulin receptor substrate 1 and insulin receptor substrate 2 in the BAT of the rats. The cold also increased glucose clearance during a glucose tolerance test. These findings support increased insulin sensitivity in the BAT. The cold-induced modulation of insulin signaling caused BAT to use energy for thermogenesis and led to an overall increase in glucose utilization [13].

In previous rat experiments, we reproducibly demonstrated that acute cold exposure (4°C for 4 hours) significantly produced higher FDG uptake in the BAT than the other tissues of rats. The FDG uptake was also much higher than that found in the non-stimulated BAT of the rats at room temperature [14]. These data led to our hypothesis that only 4-hours of cold exposure might induce the changes in the transcripts involved in the insulin receptor signaling pathway of BAT. We assumed that the more transcripts in the pathway that are significantly regulated, the more reliable the prediction of the status of the pathway would be. Since no study has focused on all of the transcripts involved in the insulin signaling pathway in the BAT of rats, we used updated rat whole genomic chips to assess the responses of all transcripts involved in the insulin signaling pathway and the associated carbohydrate metabolism in BAT of rats after 4 hours of cold exposure at 4°C.

## Materials and Methods

### Animals

Female Lewis rats (body weight 126–150 g) were purchased from Charles River Laboratories, Inc (Wilmington, MA). They were housed in the animal facilities (room temperature 22–23°C) and exposed to a 12 hour light/dark cycle at least 1 week before the start of experimentation. During this adaptation period, food and water were given ad libitum. The study was performed as part of a project approved by the animal research committee at the Johns Hopkins Medical Institutions.

### BAT activation

Female rats (n = 6) were randomly divided into two groups of three each. The rats in the control group (CTL) were kept at room temperature, 22°C. The rats in the treatment group were placed at 4°C for 4 hours [14], [15], [16]. In both groups, food was withdrawn for the 4-hour conditioning period. Animals were humanely sacrificed using CO_2_ asphyxiation. At sacrifice, the BAT in 4°C cold-treated group appeared highly perfused and was darker and redder in color than the BAT from the 22°C control group, consistent with BAT activation after 4°C exposure.

### RNA extraction and microarray-based assessment of relative transcript abundance

Interscapular BAT was dissected from each animal immediately after the animals were sacrificed and separately stored in liquid nitrogen. Total RNA was extracted from the samples with the RNeasy kit purchased from QIAGEN (Germantown, MD). The quality and quantity of the RNA were evaluated with a NanoDrop UV-Vis spectrophotometer and Agilent Bioanalyzer. The Rat Expression 230 2.0 microarray chip from Affymetrix (Santa Clara, CA) is a whole-genome array to interrogate 31,099 transcripts and variants from the rat genome, and utilized by the Microarray Core Laboratory at Johns Hopkins Medical Institutes. A total of 6 chips were used (3/group), one per rat BAT sample.

### Analysis of the microarray data and signaling pathways

To remove sources of variation of non-biological origin among arrays, the microarray raw data were first normalized by RMAexpress (Robust Multichip Average express), software [17]. The significance of gene expression levels between the cold and control groups were assessed by Spotfire (TIBCO, Somerville, MA), commercial software for gene array analysis.

The transcripts with a p-value less than 0.01 were selected for further analysis of signaling pathways. Using analytic software, Ingenuity Pathway Analysis (IPA, Ingenuity Systems, Redwood City, CA), these transcripts were grouped based on their biological functions and apparent involvement in signaling pathways. The Fisher's Exact Test was used to compute the p-value with the Benjamini-Hochberg multiple testing correction applied to control for the false discovery rate. Since most p-values from the Fisher's and Benjamini tests were very small, we present them as −log_10_ (p-value), which we term the “p-score”.

To assess the activation states of various biological processes, activation z-scores were computed based on the change in the direction of the transcripts and the interactive relationship among genes. If the changes in our data were consistent with the changes of the known activation model in the Ingenuity System, the z-scores would be positive. The z-scores would be negative if opposite occurred. In the current study, z-scores would be considered significant if they were greater than 2 (activation) or smaller than -2 (inhibition). The reports of statistical analysis, pathways, and biological processes were generated by the use of IPA (Ingenuity Systems, www.ingenuity.com, Redwood City, CA).

### Western blotting for insulin signaling

Nine rats were fasted overnight. The next morning, they were randomly put into one of three conditions: room temperature + saline (RT, nonstimulated), cold temperature + saline (4°C for 4 hours), and room temperature with insulin administration (0.75 units/kg), HumulinR from Eli Lilly, USA[18], as positive controls of insulin signaling activation. All injections (saline or insulin) were intraperitoneally administrated 10 minutes before sacrifice. The intrascapular brown adipose tissue and scapular muscles were immediately dissected and separately frozen with dry ice. At the end of the experimental condition each rat was humanely sacrificed with the method described above.

Approximately 10 mg brown adipose tissue was used for the preparation of lysate from each sample with RIPA lysis buffer (9806s, Cell Signaling, MA, USA) plus phosphatase inhibitor. Muscle lysates were prepared following the same steps.

Approximately 30 µg protein lysate per lane was loaded to NuPAGE Bis-Tris Mini Gels (MAN0003679, life tech, CA, USA). After electrophresis, the samples were blotted onto nitrocellulose membranes. The membranes were visualized by Ponceaus staining (P3504, Sigma, MO, USA) and cut into distinct strips based on the target protein's molecular weight. Rabbit primary antibodies against the insulin receptor (#3025), Irs2 (#4502), Irs1 (#2382), Akt-p (#9271), Akt-t (#9272) were obtained from Cell Signaling. Anti-Glut4 (SAB4300667), anti-β actin (A1978, Sigma), and actin (A2103, Sigma) were obtained from Sigma. Donkey anti-rabbit Ig HRP conjugate (NA9340) was obtained from Amersham (GE Healthcare, Buckinghamshire, UK). Signals were detected by enhanced chemiluminescence plus the Western Blotting Detection System (RPN2232, GE Healthcare, Buckinghamshire, U.K.).

After an overnight incubation with block buffer (TBST+5% nonfat), the primary antibodies 1∶1000 except for β-actin (1∶5000) and actin (1∶35000) were incubated with the blotted nitrocellulose membranes overnight. These processes were performed at 4°C with gentle agitation on an orbital shaker. The secondary antibodies (1∶2000 for anti-rabbit-HRP and 1∶5000 for anti-mouse-HRP) were incubated for 1 hour at room temperature with gentle agitation. The signals on Hyperfilm ECL (#28906835, GE healthcare, Buckinghamshire, UK) were scanned into a computer and analyzed with Image Studio Lite Ver 3.1 (LI-COR Corporate, NE, USA). The β-actin signal in each sample served as a denominator to normalize the insulin signaling in the current experiments. We obtained relative signal intensities for further comparisons to determine fold changes from controls. Student's t test was employed to examine the protein expression differences among groups.

## Results

### Cold-induced change in the insulin receptor signaling pathway

The top 4 altered canonical pathways in BAT responding to cold stimulation were insulin receptor signaling, protein kinase A signaling, PI3K/AKT signaling, and ERK/MAPK signaling. All of them had low false discovery rates (p<0.001).

In the insulin receptor signaling pathway with the documented 142 genes, 42 transcripts (29.6%) significantly responded to 4 hours of cold exposure. Among 42 genes, the down-regulation was 15/42 (36%) and the up-regulation was almost double, 27/42 (64%).

**Figure 1:**
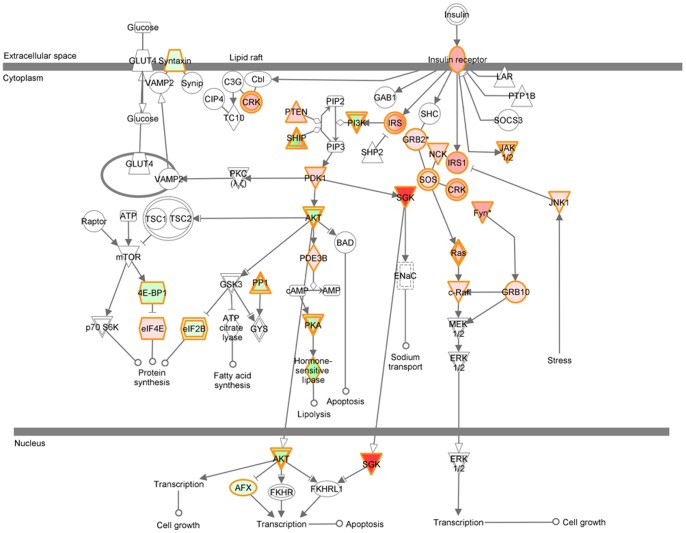
Diagram of the insulin signaling pathway modified by coding the change of transcripts in the BAT of rats responding to 4-hours of cold stimulation (4°C) as compared to the control group (22°C). Up-regulated transcripts are red (the darker red, the more up-regulation). Down-regulated ones are green (the darker green, the more down-regulation). The unchanged transcripts have no color. The signs between two components represent activation (arrow) and inhibition (bar or bar crossing arrow). Our data show that 4-hours of cold stimulation regulated multiple transcripts of the insulin signaling pathway in BAT of rats.

To visualize the relationship among the transcripts, these 42 transcripts were graphed to a modified diagram of the insulin receptor signaling pathway with red (up-regulation), green (down-regulation), or non-color (unchanged) by IPA (Figure 1). The diagram revealed concurrent up-regulation in the insulin receptor-MAPK branch, but mixed regulation in the insulin receptor-PI3K branch (Figure 1).

At the initial part of the pathway, the transcript of the insulin receptor (Insr) was up-regulated, and associated with concurrently up-regulated transcripts of insulin receptor substrate 1 (Irs1), insulin receptor substrate 2 (Irs2), and Jak1 (Janus kinase 1) (Table 1 and Figure 1). The transcripts of phosphatases, which dephosphorylate the insulin receptor, were not changed.

**Table 1:** Insulin receptor signaling pathway.

Symbol	Gene Name	LogRatio	p-value	Type(s)	Affymetrix
SGK1	serum/glucocorticoid regulated kinase 1	3.34	6.03E-05	kinase	1367802_at
IRS2	insulin receptor substrate 2	2.40	3.27E-05	enzyme	1371091_at
INPP5B	inositol polyphosphate-5-phosphatase, 75kDa	2.06	6.88E-04	phosphatase	1388502_at
FYN	FYN oncogene related to SRC, FGR, YES	1.51	6.65E-05	kinase	1373683_at
IRS1	insulin receptor substrate 1	1.42	7.73E-04	enzyme	1369771_at
INSR	insulin receptor	1.38	2.96E-03	kinase	1392043_at
KRAS	v-Ki-ras2 Kirsten rat sarcoma viral oncogene homolog	1.15	7.65E-06	enzyme	1370035_at
CRK	v-crk sarcoma virus CT10 oncogene homolog (avian)	1.07	9.14E-05	other	1392462_at
PRKAR1A	protein kinase, cAMP-dependent, regulatory, type I, alpha	1.04	5.22E-04	kinase	1386905_at
PIK3C2A	phosphatidylinositol-4-phosphate 3-kinase, catalytic subunit type 2 alpha	0.87	4.36E-04	kinase	1379433_at
PTEN	phosphatase and tensin homolog	0.80	1.30E-03	phosphatase	1370112_at
SH2B2	SH2B adaptor protein 2	0.78	2.00E-03	other	1368605_at
RRAS2	related RAS viral (r-ras) oncogene homolog 2	0.74	2.82E-03	enzyme	1382058_at
PDPK1	3-phosphoinositide dependent protein kinase-1	0.72	5.38E-03	kinase	1370052_at
NCK1	NCK adaptor protein 1	0.68	2.87E-03	kinase	1373940_at
PPP1R3D	protein phosphatase 1, regulatory subunit 3D	0.66	7.79E-03	phosphatase	1373656_at
GRB2	growth factor receptor-bound protein 2	0.53	5.95E-03	other	1368385_a_at
GRB10	growth factor receptor-bound protein 10	0.51	6.56E-03	other	1371517_at
PPP1R12A	protein phosphatase 1, regulatory subunit 12A	0.51	8.78E-03	phosphatase	1382307_at
MAPK8	mitogen-activated protein kinase 8	0.49	1.27E-03	kinase	1379612_at
SOS1	son of sevenless homolog 1 (Drosophila)	0.48	3.52E-03	other	1389710_at
EIF4E	eukaryotic translation initiation factor 4E	0.47	5.09E-03	translation regulator	1398799_at
SYNJ1	synaptojanin 1	0.43	1.35E-03	phosphatase	1370070_at
PDE3B	phosphodiesterase 3B, cGMP-inhibited	0.37	5.71E-03	enzyme	1369157_at
RAF1	v-raf-1 murine leukemia viral oncogene homolog 1	0.34	1.37E-03	kinase	1369932_a_at
PIK3CA	phosphatidylinositol-4,5-bisphosphate 3-kinase, catalytic subunit alpha	0.27	9.47E-03	kinase	1382366_at
JAK1	Janus kinase 1	0.26	1.62E-03	kinase	1383478_at
STX4	syntaxin 4	-0.16	4.38E-03	transporter	1370014_at
FOXO4	forkhead box O4	-0.26	1.47E-03	transcription regulator	1372652_at
EIF2B4	eukaryotic translation initiation factor 2B, subunit 4 delta, 67kDa	-0.28	8.86E-03	translation regulator	1386970_at
PRKACA	protein kinase, cAMP-dependent, catalytic, alpha	-0.30	7.88E-04	kinase	1371578_at
PIK3C3	phosphatidylinositol 3-kinase, catalytic subunit type 3	-0.40	1.17E-03	kinase	1369655_at
INPPL1	inositol polyphosphate phosphatase-like 1	-0.41	7.55E-03	phosphatase	1368022_at
EIF4EBP1	eukaryotic translation initiation factor 4E binding protein 1	-0.43	6.92E-03	translation regulator	1386888_at
PPP1R3C	protein phosphatase 1, regulatory subunit 3C	-0.48	1.93E-03	phosphatase	1373108_at
AKT2	v-akt murine thymoma viral oncogene homolog 2	-0.61	1.31E-03	kinase	1388765_at
PRKAG2	protein kinase, AMP-activated, gamma 2 non-catalytic subunit	-0.62	1.38E-03	kinase	1373952_at
INPP5K	inositol polyphosphate-5-phosphatase K	-0.64	1.12E-04	phosphatase	1373627_at
PRKAR2B	protein kinase, cAMP-dependent, regulatory, type II, beta	-0.68	1.53E-04	kinase	1371133_a_at
PIK3R2	phosphoinositide-3-kinase, regulatory subunit 2 (beta)	-0.71	2.44E-04	kinase	1376190_at
LIPE	lipase, hormone-sensitive	-0.73	6.29E-03	enzyme	1387132_at
INPP5F	inositol polyphosphate-5-phosphatase F	-0.85	7.41E-03	phosphatase	1389176_at

The cold-induced transcripts (p<0.01) of the insulin signaling pathway are shown above. The interactive relationships among the transcripts are shown in Figure 1. Based on the extent of the changes, the transcripts are listed from the highest to the lowest. Log Ratio  =  log2 (average cold/average control). The p-values are represented in the scientific format of Microsoft Excel (for example, 6.03E-05 = 6.03×10^−05^).

**Figure 2:**
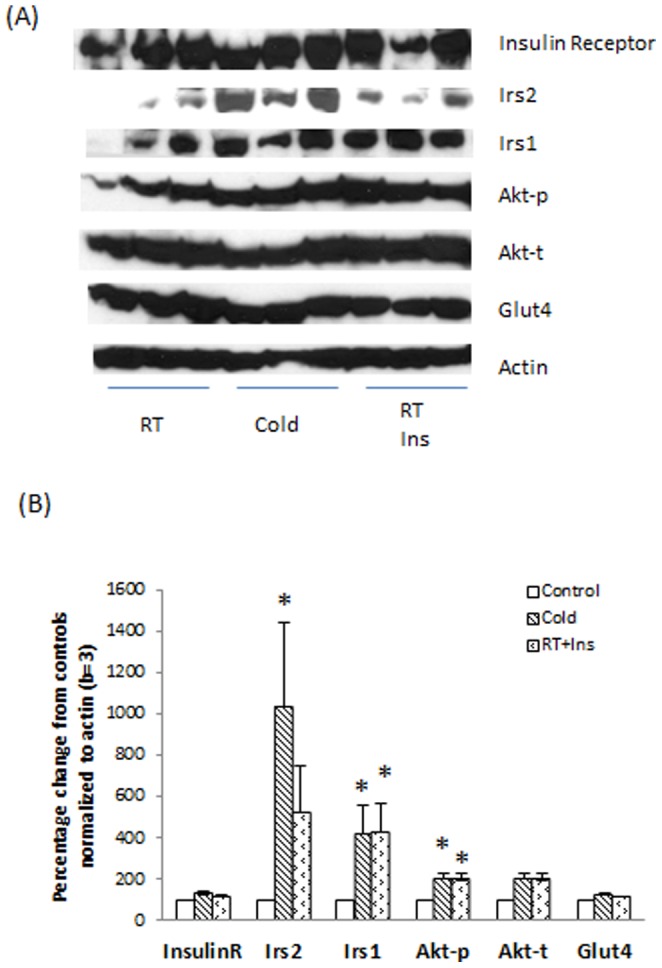
Western blot showing expression of the insulin receptor, Irs2, Irs1, Akt-p, Akt-t, Glut4, and Actin in the BAT of rats with/without stimulation. Nine rats were fasted overnight. The next morning, they were randomly exposed to one of three conditions: room temperature + saline (RT, **control**), cold temperature + saline (4°C for 4 hours, **Cold**), and room temperature with insulin administration (0.75 units/kg, **RT+ins**). The preparation of BAT lysates was performed as described in Materials and Methods. Panel A: Protein levels of Insulin receptor, Irs2, Irs1, Akt-p, Akt-t, Glut4, and Actin, analyzed and compared to controls. Panel B: Columns represent mean ± SEM protein levels determined from “A” as a percentage control values of three experiments (t-test with α = 0.05, * p<0.05). The insulin signaling pathway in the BAT of rats post the acute cold exposure was more active than controls with increased Irs2, Irs1, Akt-p post cold exposure.

In the MAPK branch, concurrent up-regulation is shown (Figure 2), including Sos1 (son of sevenless homolog 1), Nck (non-catalytic region of tyrosine kinase adaptor protein), Crk (V-Crk sarcoma virus CT10), Ras (rat sarcoma), Raf (rapidly accelerated fibrosarcoma), growth factor receptor bound protein 10 (Grb10), Fyn (FYN oncogene related to Src, Fgr, Yes) (Table 1 and Figure 1). The transcript of MAPK8 (mitogen-activated protein kinase 8)/Jnk1 was up-regulated.

We analyzed the three classes of the PI3K family, class I, II, and III. The Pik3ca (catalytic, class I) and Pik3c2a (class II) were up-regulated. The Pik3r2 (regulatory subunit 2, class I) and Pik3c3 (class III) were down-regulated. However, the sum of phosphoinositide-3-kinase (Pik3) was down-regulated since the down-regulation of Pik3r2 and Pik3c3 was greater than the up-regulation of Pik3ca and Pik3c3 (Table 1 and Figure 2). Pdk1 (3-phosphoinositide dependent protein kinase-1), a downstream kinase of PI3K, was up-regulated, associated Sgk1 (serum/glucocorticoid regulated kinase 1) up-regulation but the Pkc family was unchanged. Akt2 (v-akt murine thymoma viral oncogene homolog 2) was down-regulated in the current study. Transcripts of Pka and hormone sensitive lipase (Lipe) were down-regulated in the setting of up-regulated Pde3b (phosphodiesterase 3B, cGMP-inhibited). Overall, the over-expressed transcripts in the PI3K/AKT branch were greater than the underexpressed transcripts.

### Cold-induced gene change involved in carbohydrate metabolism

Using IPA Downstream Effects Analysis, we calculated p-values and z-scores to predict the association among the changed transcripts in our data as compared to the transcript interaction related to carbohydrate metabolism (Table 2). There were two sets of transcripts corresponding to carbohydrate metabolism. One set of transcripts is involved in the metabolism of carbohydrates (p-value 5.16×10^−10^ and had an activated z-score 2.643 giving 66% up-regulation and 34% down-regulation in 166 transcripts). The other set of transcripts is related to synthesis of carbohydrate (p-value 7.60×10^−10^ and an activated z-score of 2.556 due to 70% up-regulation and 30% down-regulation in 125 transcripts).

**Table 2:** Predicted activation state of carbohydrate metabolism after 4 hours of cold exposure.

Functions	p-Value	Predicted Activation State	Activation z-score	# Molecules
metabolism of carbohydrate	5.16E-10	Increased	2.643	136
synthesis of carbohydrate	7.60E-10	Increased	2.556	101

### Cold-induced change in glucose transporters

Glucose transporters within plasma membranes are crucial for brown adipocytes to obtain glucose for immediate utilization or storage. We determined the messenger RNA levels of glucose transporters in the brown adipose tissue of rats exposed to 4°C and 22°C for 4 hours. As expected, glucose transporter 4 (Glut4/Slc2a4) was much more abundant than glucose transporter 1 (Glut1/Slc2a1) (Table 3). Glut1 messenger RNA was up-regulated by cold exposure, p<0.001. Glut4 messenger RNA was not significantly changed by cold exposure as compared to the control.

**Table 3:** Cold-induced changes of the glucose transporters.

Symbol	Gene Title	Cold (log2)	Control (log2)	Fold-change (log2)	p-value	Affymetrix
Slc2a1	solute carrier family 2 (facilitated glucose transporter), member 1	7.69	6.85	0.84	0.001	1370848_at
Slc2a4	solute carrier family 2 (facilitated glucose transporter), member 4	12.26	12.19	0.07	0.626	1367989_at

### Cold-induced insulin signaling changes at the protein level

We measured the levels of Insr, Irs2, Irs1, Akt-p, Akt-t, Glut4, and β-actin proteins in the BAT lysates from the rats exposed to the three conditions described in Methods (Figure 2. A and B). Cold and insulin-stimulated BAT lysates appeared to have more Insr, Irs2, Irs1, Akt-p, Akt-t, and Glut4 than controls. Irs2, Irs1, and Akt-p in cold group and Irs1 and Akt-p in insulin-stimulated group were higher than controls (p<0.05).

**Figure 3:**
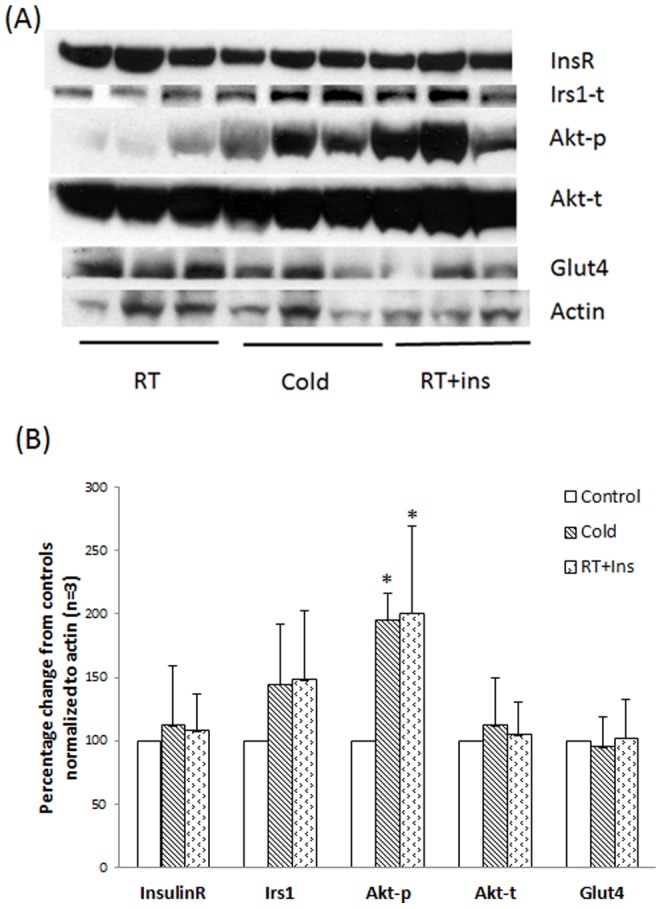
Expression of the insulin receptor, Irs1, Akt-p, Akt-t, Glut4, and Actin in the skeletal muscles of rats with/without stimulation. Nine rats were fasted overnight. The next morning, they were randomly exposed to one of three conditions: room temperature + saline (RT, **control**), cold temperature + saline (4°C for 4 hours, **Cold**), and room temperature with insulin administration (0.75 units/kg, **RT+Ins**). The preparation of BAT lysates was performed as described in Materials and Methods. Panel A: Protein levels of Insulin receptor, Irs1, Akt-p, Akt-t, Glut4, and Actin. Panel B: Columns represent mean ± SEM protein levels determined from “A” as a percentage control values of three experiments (t-test with α = 0.05, * p<0.05). The insulin signaling pathway in the muscles of rats post the acute cold exposure was likely more active than controls, as supported by increased Akt-p.

We explored the insulin signaling pathway in the skeletal muscles from the same rats which provided BAT. Since the β-actin is absent in skeletal muscles, we normalized the protein levels with total actin. Cold and insulin-stimulated skeletal muscle lysates had more Akt-p than controls (p<0.05) (Figure 3). The protein levels of Insr, Irs1, Akt-t and Glut4 were similar to the controls without significant changes. Irs2 signals in the skeletal muslces were very weak in all of conditions.

## Discussion

In the present study, we found that 4-hours of cold exposure regulated multiple transcripts involved in the insulin, PKA, PI3K/AKT and ERK/MAPK pathways in the BAT of rats. These four canonical pathways were the top pathways responding to the 4 hours of cold exposure based on their significances compared to controls. It has been documented that PKA, PI3K/AKT and ERK/MAPK signaling pathways were altered in BAT following adrenergic agonist stimulation [19], [20], [21]. Given the fact that the effect of cold exposure on the BAT is substantially mediated by the sympathetic system [1], it is not unexpected to see the changes of these three canonical pathways in the BAT of rats after the 4 hours of cold exposure.

Our data showed that the insulin signaling pathway was the most affected canonical pathway responding to 4 hours of cold exposure. Since the up-regulated transcripts were much greater than the down-regulated transcripts, this change suggested that the direction of the insulin signaling pathway was likely up. This was further supported by our western blotting data of insulin signaling and is consistent with the chronic cold exposure study of Gasparetti et al [13].

Physiologically, it is likely that the functioning brown adipose tissue in normal mammals responds to cold environments by increasing energy consumption for the short term and increasing the number of mature brown adipocytes for the long term[1]. Carbohydrates are a major energy source for a physiological response to the cold in BAT [22].

Cameron and Smith reported that cold induced the multilocular cells of BAT to lose their lipid vacuoles and led to a decrease in their size in the first 6 to 12 hours of cold exposure, but these features are restored to normal by 24 hours of cold exposure. Cell proliferation, as estimated by the DNA synthetic index method (using tritiated thymidine autoradiography), appeared in the brown fat at 1 day of cold exposure, became maximal at 4 days of cold exposure, and returned to the control level by 16 days of cold exposure [23].

Rat studies have demonstrated that cold exposure decreased insulin secretion [9], [10], [11], [13]. Improving the insulin receptor signaling pathway is likely an important mechanism for brown adipose tissue to obtain glucose more efficiently in the setting of cold-induced hypoinsulinemia [13]. The current transcriptome data matched this scenario with concurrently up-regulated crucial components in the insulin receptor signaling pathway including the insulin receptor, insulin receptor substrate 1, and insulin receptor substrate 2. Western blotting data demonstrated responses which supported our data that 4 hours of cold exposure significantly induced more Irs2 and Irs1 than controls. These data were also similar to the effects of insulin on the BAT of rats kept at the room temperature. Even though Gasparetti et al did not record a significant increase in the protein level of the insulin receptor and Irs1, they found that the protein level of Irs2 increased significantly.

Our data (4°C for 4 hours) suggested that the balance between kinase (Insr upregulation) and protein tyrosine phosphatases (Lar and Ptp1b) transcripts favored phosphorylation. This corresponded to Gasparetti et al findings that 8 days of cold exposure significantly enhanced the effects of insulin on phosphorylation of the insulin receptor, insulin receptor substrate 1, and insulin receptor substrate 2 in the brown adipose tissue of male rats [13].

The PI3K branch is well- known for regulating glucose metabolism in adipose tissue while insulin binds to its receptors [24]. Akt is activated by phosphorylation via Irs2/PI3K and Irs1/PI3k in adipose tissue [24]. Our data showed that the cold condition significantly upregulated Pdk1 mRNA level, and enhanced Akt-p at the protein level. As compared to the positive control of activating insulin signaling by administrating a dose of insulin to rats, we found that 4 hours of cold stimulation had similar effects as insulin on the BAT of rats. This finding supported cold stimulating the insulin signaling pathway similar to the effect of this insulin dose on BAT.

We demonstrated that the current cold condition up-regulated the MAPK branch. The underlying biology is most likely related to a marked reduction in the lipid storage in the brown adipose tissue as shown in our previous study after an acute cold expose [14]. Other data support this [23], [25], [26]. After lipid depletion the brown adipose tissue has to depend on exogenous energy sources such as glucose and fatty acids to maintain the thermogenesis [22].

Our data also demonstrated that cold exposure up-regulated the multiple transcripts of carbohydrate metabolism. These changes are consistent with prior studies showing the cold induced an increase in glucose uptake in brown adipose tissue [3], [4], [6], [9], [11], [12].

Shivering is a common phenomenon in mice, particularly when first moving them from room temperature to an extreme cold environment such as 4°C [27]. Mizuma et al reported that a cold ambient temperature significantly increased FDG uptake in muscles and BAT of mice (18°C vs. 38°C) due to shivering [28]. Using a FDG micro-PET scanner, Wang et al demonstrated that intense FDG activity accumulated in their BAT after the experimental mice had been put in a cold room (4°C) for 5 hours (FDG was injected after 4 hours and the mice sacrificed one hour later) but no obvious FDG activity was identified in muscles [29].

In our previous experiments, 4 hours of cold exposure (4°C) did not significantly enhance the FDG uptake in the muscles of rats one-hour post FDG injection as compared to controls (p>0.05) [14]. This suggested that the 4°C experimental condition might have been easily adapted by rats or mice through a short-period of shivering to non-shivering thermogenesis or no shivering thermogenesis at all. The Akt-p levels in the skeletal muscles of the rats stimulated by cold and insulin were elevated, and suggested that insulin signaling likely was occurring in the skeletal muscles. The underling mechanism of the cold exposure to increase the insulin signaling pathway in BAT and muscles should be further explored.

Glucose transporters, often the rate-limiting step for glucose clearance, presented a different response to cold exposure. In our short term cold exposure, glucose transporter 4 messenger RNA or protein, the predominant subtype in brown adipose tissue, was not increased as was reported in the long term cold exposure experiments [13], [30], [31]. No significant Glut4 increase in the BAT and muscle lysates from cold and insulin stimulation was detected by western blotting. Based on increased Akt-p by cold and insulin stimulation, Glut4 translocation had a high likelihood of occurrence. We observed significantly up-regulated glucose transporter 1 (Glut1), consistent with a previous report [14]. The mechanism of regulating glucose transporter 1 in brown adipose tissue may be related to the overexpression of the Ras family [32], [33]or via β3 adrenergic receptor pathways [34].

Using primary brown adipocytes, Dallner et al reported that mature brown adipocytes had more Glut4 and less Glut1 than premature brown adipocytes [34]. Activating β3 adrenergic receptors with norepinephrine increased Glut1 (peak at 2 hours post activation) and decreased Glut4 expression (nadir at 5 hours post activation) [34]. This is similar to our cold-induced Glut1 change in the BAT of rats, suggesting possible β3 adrenergic receptor involvement of Glut1 upregulation. Dallner et al also found that Actinomycin D abolished the late phase of glucose uptake, but it did not abolish the early phase of glucose uptake in brown adipocytes stimulated by β3 adrenergic receptors and it did not affect insulin-induced glucose uptake in brown adipocytes [34].

Gasparetti et al found that the effects of cold on insulin signaling and glucose uptake in the BAT of rats were mediated by β3-dependent and –independent mechanisms [35]. They demonstrated that cold stimulation, BRL37344 (a β3 agonist) and SR59230A (a β3 antagonist) increased Glut4 protein levels in the BAT of rats [35]. However, there was a counteractive effect of the combination of cold stimulation and BRL37344 on Glut4 expression, which suggested that the cold condition desensitized the BRL37344 signal transduction in the BAT of rats [35]. Gasparetti's group did not assess Glut1 under their conditions. Further evaluation of glucose transporter efficacy under different conditions might help to illustrate the mechanism of cold-induced glucose utilization in the BAT.

## Conclusion

This study demonstrated that 4-hours of cold exposure stimulated the insulin signaling pathways in the BAT of rats. Further exploration of the insulin signaling in the BAT of diabetic and obese models under cold regimens will provide more information for potential clinical translation.
